# Montmorillonite-based essential oil carrier and its effects on non-target species: an environmental perspective on its risk assessment

**DOI:** 10.3389/ftox.2025.1696913

**Published:** 2025-10-30

**Authors:** Sofia Machado, Catarina Ganilho, Tatiana Andreani, Rose Marie O. F. Sousa, Artur Ribeiro, Ruth Pereira

**Affiliations:** ^1^ GreenUPorto, Sustainable Agrifood Production Research Centre and INOV4AGRO, Department of Biology, Faculty of Sciences, University of Porto, Porto, Portugal; ^2^ Chemistry Research Centre (CIQUP) and Institute of Molecular Sciences (IMS), Department of Chemistry and Biochemistry, Faculty of Sciences, University of Porto, Porto, Portugal; ^3^ CEB, Centre of Biological Engineering, University of Minho, Braga, Portugal; ^4^ LABBELS- Associate Laboratory, Braga, Portugal

**Keywords:** *Satureja montana*, nanoclay, biopesticides, ecotoxicology, non-target organisms

## Abstract

**Introduction:**

Essential oils (EO), rich in bioactive metabolites with biocidal activity, present great potential for agricultural applications as new biopesticides. However, their high volatility and sensitivity to environmental conditions limits their application. To address these limitations, nanotechnology-based formulations have been developed, incorporating EO into natural clays such as montmorillonite (MMT). Due to its colloidal properties, high adsorption capacity, and modifiable surface, MMT serves as an effective carrier for stabilizing EO while controlling their release. Besides aiming to enhance EO efficacy, these MMT-based formulations also aim to minimize EO toxicity to non-target organisms.

**Materials and methods:**

In this study, the toxicity of *Satureja montana* EO (SM EO), of its dispersant agent Tween 20^®^, of the MMT nanoclay and of the nanoclay-EO formulation was evaluated using aquatic (*Aliivibrio fischeri*, *Raphidocelis subcapitata*, *Lemna minor* and *Daphnia magna*), and terrestrial (*Folsomia candida* and soil microbiota) non-target model organisms, following standard protocols.

**Results:**

Among the tested species, *R. subcapitata* and *D. magna*, exhibited the highest sensitivity, with *D. magna* showing an EC_50_ of 0.011 mg mL^-1^ and a complete growth inhibition being observed for *R. subcapitata* at concentrations ≥0.021 mg mL^-1^, for the nanoclay-EO formulation. *F. candida* reproduction was also significantly reduced for all tested concentrations of the nanoclay-EO formulation. In contrast, it was observed a stimulatory effect on soil microbial activity particularly for dehydrogenase and acid phosphatase enzymes.

**Discussion:**

These findings suggest that the nanoclay-EO formulation did not reduce the toxicity of SM EO, and in some cases, may even raise ecotoxicological concerns, particularly for aquatic and soil invertebrates. This study highlights the importance of detailed ecotoxicological evaluations of biopesticide formulations based on plant-based and materials as essential oils, and other natural materials, as they cannot be assumed as safe compounds. To the best of our knowledge ecotoxicological data is limited for most of the EO including some that already in the market. Based on these results, the concentrations to be tested for efficacy against target organism (safe to non-target organism) should be lower than 0.007 mg mL^-1^.

## 1 Introduction

According to a definition of the European Federation of Essential Oils (EFEO), essential oil (EO) is “a volatile part of a natural product, which can be obtained by distillation, steam distillation, or expression in the case of citrus fruits” (Recommendation on the definition of essential oil, 2021). EO are rich in bioactive compounds such as volatile terpenoids and phenolic compounds, which exhibit physiochemical (e.g., antioxidant activity) and biological properties (e.g., bactericidal and fungicidal activity). These properties make EO promising candidates for agriculture applications, particularly as biopesticides to prevent and control plant diseases and pests (Essifi et al., 2022). However, due to their high volatility and low photostability, EO are commonly susceptible to environmental factors such as sunlight and temperature (Duarte et al., 2017). For example, clove essential oil and thymol (one of the major components of *Satureja montana* EO), exhibit DT_50_ values (time required for degradation of 50% of the compound) of approximately 1.08 and 1 day, respectively, reinforcing their sensitivity to degradation (Lewis et al., 2016) (http://sitem.herts.ac.uk/aeru/bpdb/atoz_bpdb3.htm#C). Although DT_50_ values under field conditions are not reported, they are expected to be even lower.

Over the past decades, a significant number of plant-derived nanopesticides have been demonstrated to be effective at controlling pests and diseases compared to traditional forms of pesticides (Gahukar and Das, 2020). The effectiveness of nanobiopesticides depend on the ability of nanomaterials to preserve the stability and bioactivity of botanical compounds. This includes protecting them from biotic (e.g., microbial activity) and abiotic factors (e.g., temperature, light), while enabling their controlled release at the target organisms (An et al., 2022). Although comprehensive (eco)toxicological evaluations of these novel formulations remain limited, nanobiopesticides are also expected to exhibit reduced toxic effects on non-target organisms (de Oliveira et al., 2014) (and unpublished data from the authors).

A wide range of nanomaterials can be developed from natural compounds, including clay minerals. These minerals are commonly classified into four types that vary mainly in their layered structure, specifically the arrangement of tetrahedral and octahedral sheets. The kaolinite group consists of 1:1 clay mineral that have one tetrahedral and one octahedral sheet per clay layer. The smectite group includes 2:1 clay mineral characterized by two tetrahedral sheets sandwiching one octahedral sheet. The illite group, shares the same layer structure as smectite but with a higher interlayer charged interspace. Lastly, the chlorite group, consists of 2:1:1 clay mineral composed of an octahedral sheet adjacent to a 2:1 layer (Uddin, 2018). The montmorillonite (MMT) clay is a member of the smectite group, a 2:1 clay, featuring a central alumina octahedral sheet sandwiched between two silica tetrahedral sheets (Yaghmaeiyan et al., 2022). MMT has been reported as a suitable material for encapsulating active substances (a.s.), due to its colloidal nature, strong adsorption power, and easily modifiable surfaces, allowing compounds photostabilisation and controlled release (Margulies et al., 1985). The intercalation of active substances into montmorillonite layers, provides protection against environmentally fast degradation while, the intermolecular pressures can enable a sustained and low release resulting in prolonged bioactivity (Wanyika, 2014). Therefore, MMT has potential to be used as an effective delivery system. As reported by Essifi et al. (2022), MMT clays were successfully loaded with thyme EO, thymol, and carvacrol, showing an extended controlled release and protection of these active substance against the environmental conditions (Essifi et al., 2022). Similarly, Oliveira-Pinto et al. (2022a) developed MMT-based nanoclay formulations loaded with *S. montana* EO which exhibited antibacterial activity (0.4 mg mL^-1^) against *Xanthomonas euvesicatoria* in tomato plants (*Solanum lycopersicum*) (Oliveira-Pinto et al., 2022a). In addition to the EO antibacterial properties, the MMT nanocarrier itself also has shown to have antimicrobial properties, further supporting its use as a pesticide formulation component (Oliveira-Pinto et al., 2022b; Wang and Phillips, 2023).

Although both EO and clays are of natural origin, it is crucial to assess their safety to non-target organisms and the environment when explored in the development of biopesticides. Furthermore, due the unique properties of nanobiopesticides, it is vital to develop effective formulations and generate data to support regulatory frameworks. According to the Biopesticide Database, mentioned above, there is few or no information available regarding the ecotoxicity of biopesticides of botanical origin in non-target organisms or their environmental fate. In cases where ecotoxicological data is reported, information is usually limited and only two or three non-target species were used for testing and generating data. Therefore, the majority of the plant-based biopesticides are not approved, in most of the European Member States (Lewis et al., 2016).

In the present study, a nanobiopesticides formulation based on MMT nanoclay encapsulating *S. montana* (SM) EO was developed, and its environmental safety was evaluated following well-established ecotoxicological standard tests. Different non-target aquatic (the bacteria *Aliivibrio fischeri*, the microalgae *Rhaphidocelis subcapitata*, the macrophyte *Lemna minor* and the crustacean *Daphnia magna*) and terrestrial (springtails *F. candida*) model organisms were used. In addition, the formulation’s effects on key soil microbial parameters were also assessed to evaluate potential impacts on soil microbial activity and biogeochemical cycles. The aquatic test species selected reflect different trophic levels of the freshwater ecosystem, while the soil invertebrate *Folsomia candida* was chosen for its ecological relevance in soil compartment, inhabiting both soil litter and the soil surface.

To the best of our knowledge, this is the first study regarding the evaluation of the environmental safety of MMT nanoclay loaded with SM EO (herein mentioned as nanoclay-EO formulation). The individual contributions of the formulation components on test species were also investigated. This study therefore provides important and novel insights into the safe dosage range of the nanoclay-EO formulation for non-target species, offering critical information for its subsequent evaluation in pest and disease control.

## 2 Materials and methods

### 2.1 Test materials

Montmorillonite (Al_2_H_2_O_12_Si_4_, M = 360.307 g mol^-1^) was purchased from Fisher Scientific and Tween 20^®^ was obtained from MERCK^®^. The *S. montana* EO (winter savory) flowering tops was purchased from Florihana Distillerie (Les Grands Prés, France) (https://www.florihana.com/en/).

### 2.2 Synthesis of Nanoclay-EO formulation

**FIGURE 1:**
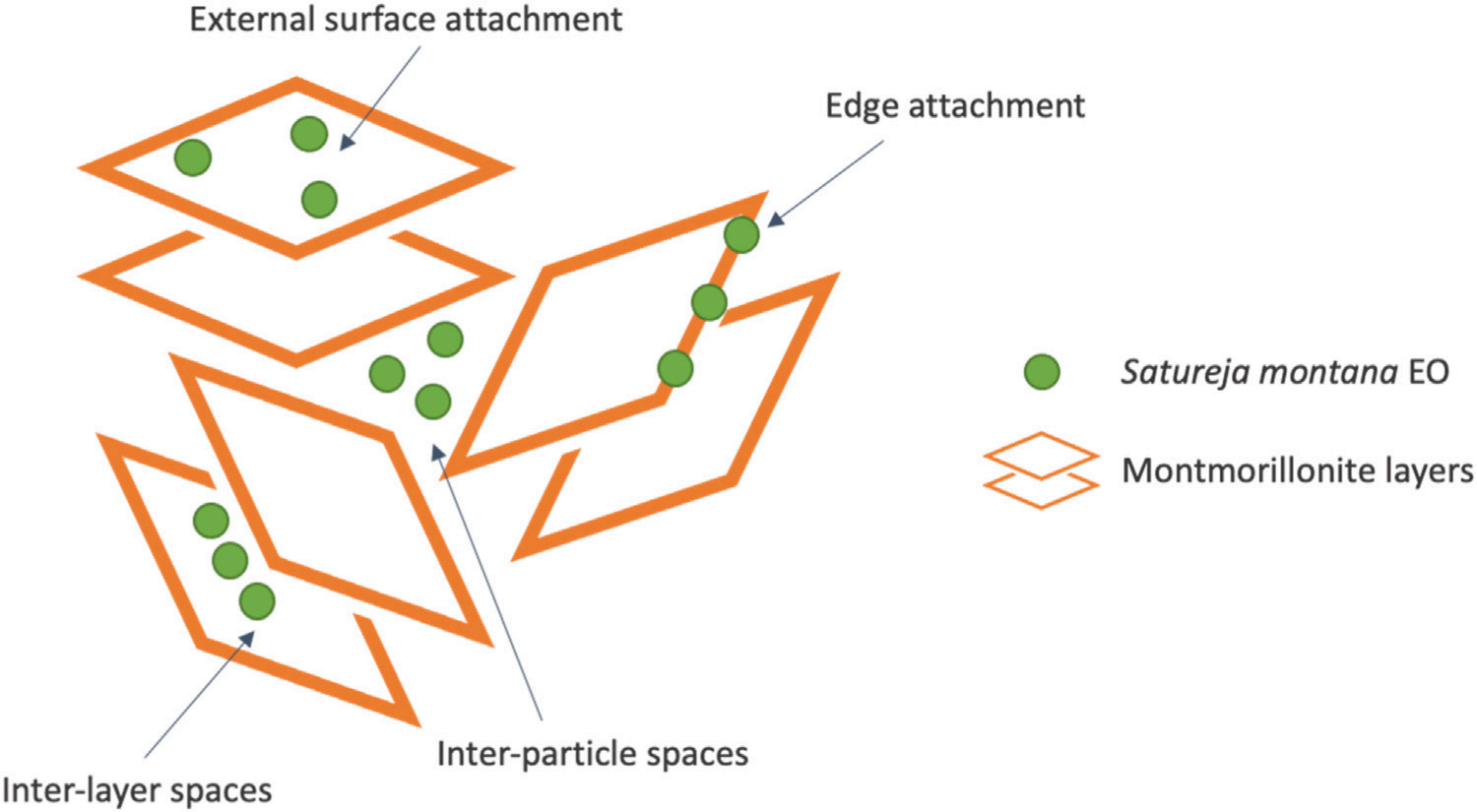
Schematic representation of the nanoclay-EO formulation.

Based on a previous study demonstrating the bactericidal and fungicidal activity of nanoclay loaded with *S*. *montana* EO at a concentration of 0.4 mg mL^-1^ (Oliveira-Pinto et al., 2022a), the same formulation was reproduced for the present study. A MMT suspension was prepared in distilled water (1% (w/v)). Then, a simple EO emulsion was prepared by mixing 3.63 μL of Tween 20^®^ (ρ = 1.10 g cm^-3^) as an emulsifier to disperse 3.92 μL of *S. montana* EO (ρ = 1.02 g cm^-3^). Afterwards, the MMT suspension was gradually mixed with the emulsion until the final volume of 10 mL was reached. So, the final concentrations of SM EO, Tween 20^®^ and of MMT in this stock suspension were 0.4 mg mL^-1^, 0.4 mg mL^-1^ and 10 mg mL^-1^, respectively. The mixture was vortexed at maximum speed for 2 min and then subjected to an ultrasonic bath (3 cycles of 3 min with 10 s pause between cycles), to facilitate the adsorption of the EO onto the nanoclay, both in the clay’s edges and in the inter-layer spaces, as schematically illustrated in Figure 1. This adsorption is driven by the negatively charged surface of montmorillonite which can interact with the EO molecules, that often contain polar groups like hydroxyls. These interactions, along with hydrogen bonding, promote the insertion of EO molecules between the clay layers (Essifi et al., 2022; Uddin et al., 2024).

### 2.3 Ecotoxicological evaluation

In the ecotoxicological tests, the test species were exposed first to the highest concentration of each component [Tween 20^®^ (0.2 mg mL^-1^), Tween 20^®^ (0.2 mg mL^-1^) + SM EO (0.2 mg mL^-1^) and MMT (5 mg mL^-1^)] corresponding to the highest concentration tested for the nanoclay-EO formulation (except for the Microtox® test). Additionally, a range of concentrations of the nanoclay-EO formulation were tested simultaneously (herein described). Later, a second exposure was performed to assess a range of concentrations of each individual component corresponding to the same range of concentrations that are present in the nanoclay-EO formulation (herein described).

#### 2.3.1 Microtox^®^ bioluminescence test

The test with *Aliivibrio fischeri* (freeze-dried NRRL strain number B11177, commercially purchased from ModernWater) was performed using the Microtox^®^ 500 Analyzer (Modernwater, Newcastle, DE, United States) following the 81.9% basic test protocol from AZUR (ISO 2007) to assess the toxicity of the nanoclay-EO formulation and its components (Azur Environmental, 1995).

*A. fischeri* was exposed to: Microtox diluent (control, CTL), Tween 20^®^, MMT, SM EO + Tween 20^®^ and the nanoclay-EO formulation (composed by SM EO + Tween 20^®^ + MMT). All were tested at a range of concentrations: 0.001, 0.003, 0.005, 0.010, 0.020, 0.041, 0.082, 0.164 and 0.328 mg mL^-1^ for Tween 20^®^, SM EO + Tween 20^®^ and nanoclay-EO formulation. The MMT suspension was tested at the concentrations of 0.03, 0.06, 0.13, 0.27, 0.51, 1.02, 2.04, 4.09 and 8.19 mg mL^-1^. The Microtox test uses a dilution factor of two and it also considers the addition of the osmotic adjustment solution, and therefore the various concentrations tested will be slightly different from the ones tested for the other aquatic test species that will be presented next. Bioluminescence emitted by the bacteria was measured before and after 5, 15, and 30 min of exposure to each concentration. For each time point, the MicrotoxOmni software automatically adjusted a probit model to data and estimated the EC_50_, along with their 95% confidence intervals. EC_50_ represents the concentration of the sample that causes an inhibition of 50% in bacteria bioluminescence. When it was not possible to calculate it, the highest effect (HE) percentage (%) is presented.

#### 2.3.2 *Raphidocelis subcapitata* growth inhibition test

The toxicity of the nanoclay-EO formulation and its individual components to freshwater microalgae was tested following the standard OECD 201 protocol (OECD, 2011), which assesses toxicological effects on microalgae growth. *Raphidocelis subcapitata*, a microalga with a sickle-like morphology known for its high sensitivity to different hazardous substances, such as organic substances (Ma et al., 2006) and metals (Gebara et al., 2020; Machado and Soares, 2024), was selected as the model used to represent freshwater producers. The microalga inoculum was obtained from a culture maintained in the laboratory at a constant temperature of 21 °C ± 2 °C, continuous lighting (7,000 lux) in Woods Hole MBL medium supplemented with vitamins [thiamine (vit. B1), biotin (vit. B7), cyanocobalamin (vit. B12)] in accordance with, OECD 201 guidelines and, maintained under constant stirring. The microalga was exposed in sterile 24-well plates with five replicates per test concentration, plus three replicates for the control (Woods Hole MBL medium). In each well, 900 μL of the nanoclay-EO formulation or of its components diluted with MBL to the desired concentrations was added, followed by the addition of 100 μL of algae inoculum to achieve an initial cell density of 10^4^ cells mL^-1^. The microplates were maintained for 72 h under the same conditions used for culturing.

The treatments tested included: Woods Hole MBL medium (control, CTL), Tween 20^®^, MMT, SM EO + Tween 20^®^, and the nanoclay-EO formulation. All were tested at a range of different concentrations (0.004, 0.007, 0.012, 0.021, 0.037, 0.065, 0.114 and 0.200 mg mL^-1^). Except for MMT that was tested at concentrations of 0.10, 0.17, 0.30, 0.53, 0.93, 1.63, 2.86 and 5 mg mL^-1^. After 72 h of exposure, cells density (cells mL^-1^) in each well was determined by counting cells using a Neubauer chamber under an optical microscope. These counts were used to calculate the microalgae growth rate relative to the control for each treatment. The results are expressed as daily growth rate.

#### 2.3.3 *Lemna minor* growth inhibition test

The effect of the nanoclay-EO formulation and its individual components on freshwater aquatic plants was evaluated using the macrophyte *Lemna minor*, an extensively used model species for ecotoxicity testing (Lakatos et al., 1993; Radić et al., 2011). The assay followed the OECD 221 protocol (OECD, 2006).

*L. minor* cultures were allowed to grow exponentially (24 °C ± 2 °C and continuous lighting at 7,000 lux) in Steinberg medium (OECD 221). The test was conducted in 6-well microplates, with three plants per well presenting three fronds each. Plants were exposed for 7 days under the same culture conditions to the following treatments: Steinberg medium (control, CTL), Tween 20^®^, MMT, SM EO + Tween 20^®^, and the nanoclay-EO formulation all at a range of different concentrations (0.004, 0.007, 0.012, 0.021, 0.037, 0.065, 0.114 and 0.200 mg mL^-1^). The exception was once again the MMT suspension that was tested at 0.10, 0.17, 0.30, 0.53, 0.93, 1.63, 2.86 and 5 mg mL^-1^. All treatments were diluted with Steinberg medium, and each treatment was tested in triplicate wells, along with the control. After 7 days of exposure, all fronds from each well were collected, counted, and dried at 60 °C to a constant weight. The number of fronds and corresponding dry biomass (dry weight) were recorded per well, and the average growth rate was calculated based on these two parameters. The results are expressed as daily growth rate.

#### 2.3.4 *Daphnia magna* immobilization test

An acute toxicity test was conducted to assess the toxicity of the nanoclay-EO formulation and its individual components to the cladoceran *Daphnia magna* Straus, a representative species of primary consumers of freshwater lentic trophic chains, following the test guideline OECD 202 (OECD, 2004).

Cultures are maintained in laboratory in 1 L glass flasks with approximately 30 organisms per 800 mL of ASTM hard water medium (OECD 202), supplemented with an organic extract of *Ascophyllum nodosum* (CAS no 84775–78–0), and vitamins [thiamine (vit. B1), biotin (vit. B7) and cyanocobalamin (vit. B12)]. The cladocerans are fed every other day with a suspension of green microalgae *R. subcapitata* (1.5 × 10^5^ cells mL^-1^) and maintained at 20 °C ± 2 °C with a 16-h light/8-h dark cycle. Neonates less than 24 h old, from the third to the fifth brood of synchronized cultures, were exposed for 48 h to: ASTM medium (control, CTL), Tween 20^®^, MMT, SM EO + Tween 20^®^, and the nanoclay-EO formulation all at range of different concentrations (0.004, 0.007, 0.012, 0.021, 0.037, 0.065, 0.114 and 0.2 mg mL^-1^), for 48 h. In the case of the individual components, only the highest concentration was tested. Four replicates per CTL and treatment, each containing five neonates in 25 mL final volume, were tested. All dilutions were prepared with ASTM hardwater medium. Immobilization was monitored visually after 24 h and 48 h of exposure, recording the number of immobilized organisms. The results are presented as percentage of immobilized individuals, and the EC_50_ was estimated for the 48 h exposure after adjusting a Probit linear regression model to the data.

#### 2.3.5 *Folsomia candida* reproduction test

The aim of this test was to evaluate whether the nanoclay-EO formulation and its individual components affect the reproduction of the collembolan *Folsomia candida*, following the test guideline OECD 232 (OECD, 2016). All test organisms were obtained from synchronized laboratorial cultures maintained in plastic containers with a mixture composed of moisten plaster of Paris and charcoal 8:1 (m: m), under controlled conditions (20 °C ± 2 °C, 16:8 h light: dark cycles and a light intensity of 600 lux). Cultures were fed weekly with hydrated yeast granules. Test organisms of homogenous age (9–12 days old) were collected from synchronized cultures. The assays were performed in small plastic containers with 30 g of OECD artificial soil (prepared according to OECD 232 (OECD, 2016)).

Stock solutions of each formulation were diluted to varying concentrations using the amount of water needed to bring the maximum soil Water Holding Capacity (WHCmax) to 50%. Ten *F. candida* individuals were exposed for 28 days to the following treatments: water (control, CTL), Tween 20^®^, MMT, SM EO + Tween 20^®^, and the nanoclay-EO formulation at different concentrations mixed with OECD artificial soil (0.07, 0.13, 0.20, 0.27, 0.47, 0.67 and 1 mL kg^-1^; see Supplementary Table S1 for w/w concentrations). When each component was tested at different concentrations, the concentrations used are the same as for the nanoclay-EO formulation. Each treatment was tested in five replicates. Food (dry yeast) was added once a week, and the test was conducted under the same conditions as described for culture maintenance. After 28 days of exposure, the soil was gently mixed, and the plastic containers were filled with water, and the content was transferred to larger plastic pots. A few drops of China ink were added to enhance contrast, and a photo was taken to the water surface with collembola floating. The number of adults and juveniles in each pot were counted using the free software ImageJ version 1.53k (Wayne Rasband and contributors, National Institutes of Health, Maryland, United States of America–Java 1.8.0_172 (64-bit) – http://imagej.nih.gov/ij).

#### 2.3.6 Soil microbial parameters

The impact of the formulations and their components on the activity of the soil microbial community was assessed by measuring the activity of soil dehydrogenases, acid phosphatases and arylsulfatase enzymes, as well as nitrogen mineralization. For this purpose, a natural soil was used, collected from the topsoil layer, between 0 and 20 cm of the agricultural fields of the GreenUPorto Research Centre, at Vairão (Vila do Conde, Portugal). This soil is characterized by a pH of 5.39 (H_2_O) and 6.46 (KCl 1M), a conductivity of 0.77 m cm^-1^, an organic matter content of 5.21% and a water-holding capacity of 43% (Ganilho et al., 2022). This soil has not been under agriculture production and weeds and/or spontaneous vegetation are mechanically controlled at least in the last 30 years. The batch of soil collected was sieved through a 2 mm mesh size before being used. The formulations’ stock suspensions were diluted to varying concentrations using the amount of water required to adjust the soil WHCmax to 80%. The treatments and the range of concentrations tested were the ones described for the collembolan’s reproduction test. For each treatment, three replicates with 100 g of fresh and sieved soil each were prepared and incubated at 20 °C ± 2 °C and at a 16 h L: 8 h D photoperiod, for 30 days. The control was only moistened with deionized water. Flasks were covered with perforated parafilm. Every 3 days during this time, each replicate was weighted, and when needed the initial weight was re-established with deionized water. Following the 30-day exposure period, six sub-replicas (3 samples +3 blank tubes) for every exposure replication and soil microbial parameter were prepared by weighting 1 g of soil to Falcon tubes, which were stored at −20 °C for a maximum of 1 month before analysis.

For the analysis of the dehydrogenase activity (Robinson et al., 1997), soil samples were incubated at 40 °C for 24 h, at a pH of 7.6, with a solution of triphenyltetrazolium chloride (TTC) (10 g L^−1^) in Tris buffer 0.1 M, whereas the three blank tubes were incubated only with Tris buffer 0.1 M. Afterwards, acetone was used to extract the triphenylformazan (TPF), formed by TTC reduction, and a complex with a pink hue was obtained. For the extraction, the tubes were placed in an orbital shaker for 2h, in the dark. Then, the tubes were centrifuged at 3,000 rpm for 4 min (Eppendorf 5,810/5810R). Afterwards, the absorbance was measured spectrophotometrically at 546 nm. The activity of the dehydrogenase enzymes was estimated using a TPF standard curve (0, 3.33, 6.67, 10, 16.67 and 33.33 µg TPF mL^-1^) in acetone, and the results were represented in µg TPF g^−1^dm (% dry matter) h^−1^.

For measuring the activity of acid phosphatases (Eivazi and Tabatabai, 1977), soil samples were incubated with a p-nitrophenylphosphate solution (11.5 mM) in the Modified Universal Buffer (MUB) at pH 6.5 for 1h, at 37 °C. The blank tubes were incubated only with MUB. Then, the tubes were centrifuged at 3,000 rpm for 4 min (Eppendorf 5,810/5810R). Afterwards, using sodium hydroxide (0.5 M), the p-nitrophenol (pNP) produced by the phosphomonoesterase’s activity was extracted, generating a yellow complex whose absorbance was measured at 400 nm. The acid phosphatase enzyme activity was estimated using a p-nitrophenol standard curve (0, 1.5, 3, 6, 12 and 24 µg pNP mL^-1^), and the results are presented in µg pNP g^−1^ dm h^−1^.

For arylsulfatase activity analysis (Tabatabai and Bremner, 1969), soil samples were incubated at 37 °C for 1 h at a pH of 5.8 in a sodium acetate trihydrate buffer 0.5 M containing a p-nitrophenylsulfate solution (0.02 M). Only sodium acetate trihydrate buffer (0.5 M), at a pH of 5.8, was added to the blank tubes. Then, the tubes were centrifuged at 3,000 rpm for 4 min (Eppendorf 5,810/5810R). The yellow-coloured complex that resulted from extracting the nitrophenol (pNP) produced by the arylsulfatase activity, using NaOH 0.5 M, was measured at 420 nm. The arylsulfatase enzyme activity was estimated using a p-nitrophenol standard curve (0, 10, 20, 30, 40 and 50 µg pNP mL), and the results are presented in µg pNP g^−1^ dm h^−1^.

To assess nitrogen mineralization (Berg and Rosswall, 1985), soil samples and blanks were incubated with deionized water, at 40 °C, for 7 days. The organic forms of nitrogen were converted throughout this procedure into inorganic forms, including NH_4_^+^, which could be detected after extraction with KCl 2 M. Then, the tubes were centrifuged at 3,000 rpm for 4 min (Eppendorf 5,810/5810R). Following the ammonia-sodium salicylate and sodium nitroprusside reaction in the presence of sodium dichloroisocyanurate, a green complex was produced, and its absorbance measured at 690 nm. Nitrogen mineralization was then estimated using a NH_4_^+^-standard curve (0, 0.1, 0.2, 0.4, 0.8 and 1.6 µg NH_4_^+^ mL^-1^). The results were expressed as µg NH_4_^+^ g^−1^ dm d^−1^.

## 3 Statistical analysis

A One-Way ANOVA was performed to evaluate the effects of the treatments on the test species and on soil microbial parameters (level of significance p < 0.05). When a significant effect was recorded, the multiple comparison Dunnet test was applied to look for significant differences between the treatments and the control. The ANOVA assumptions of normality and homoscedasticity of variances were verified using the Bartlett’s and Shapiro-Wilk’s tests, respectively. If these assumptions were not verified, the Kruskal–Wallis non-parametric test was used (Supplementary Table S2). In the case of *D. magna* Immobilization test, EC_50_ for immobilization was estimated for the 48 h exposure after adjusting a Probit linear regression model to the data.

## 4 Results and discussion

### 4.1 Ecotoxicological evaluation on the aquatic organisms

The nanoclay-EO formulation did not reduce the toxicity of the *S. montana* EO to *A. fischeri* (Table 1). The formulation remained highly toxic at all concentrations tested, despite the EO being conjugated with the MMT. The observed toxicity of the formulation is attributed mainly to the EO, since there is only toxicity when the EO is present: in the SM EO conjugated with Tween 20^®^ and in the formulation. The toxic effect is probably due to the presence of phenolic compounds (e.g., thymol and carvacrol) (Pino-Otín et al., 2022) to which *A. fischeri* seems to be extremely sensitive (Abbas et al., 2018). When the MMT and the Tween 20^®^ were tested alone, these components of the nanoclay-EO formulation displayed low to no toxicity towards the bacteria (Table 1).

**TABLE 1:** EC_50_ values, representing the concentration of each tested suspension that caused a 20% bioluminescence inhibition of *Aliivibrio fischeri* after 30 min of exposure.

	Tween 20^®^	MMT	SM EO + T20	Nanoclay-EO formulation
EC_50_ (mg mL^-1^)	>0.328	>8.190	<0.001	<0.001
Highest Effect (%)	27%	9.7%	-	-

SM EO- *S. montana* EO; MMT- Montmorillonite; T20-Tween20^®^. EC_50_ values were higher than the highest tested concentration when the highest effect recorded was lower than 50% for all the concentrations tested. EC_50_ values were lower than the lowest concentration tested, when the effect registered at the lowest concentration was already greater than 50%.

**FIGURE 2:**
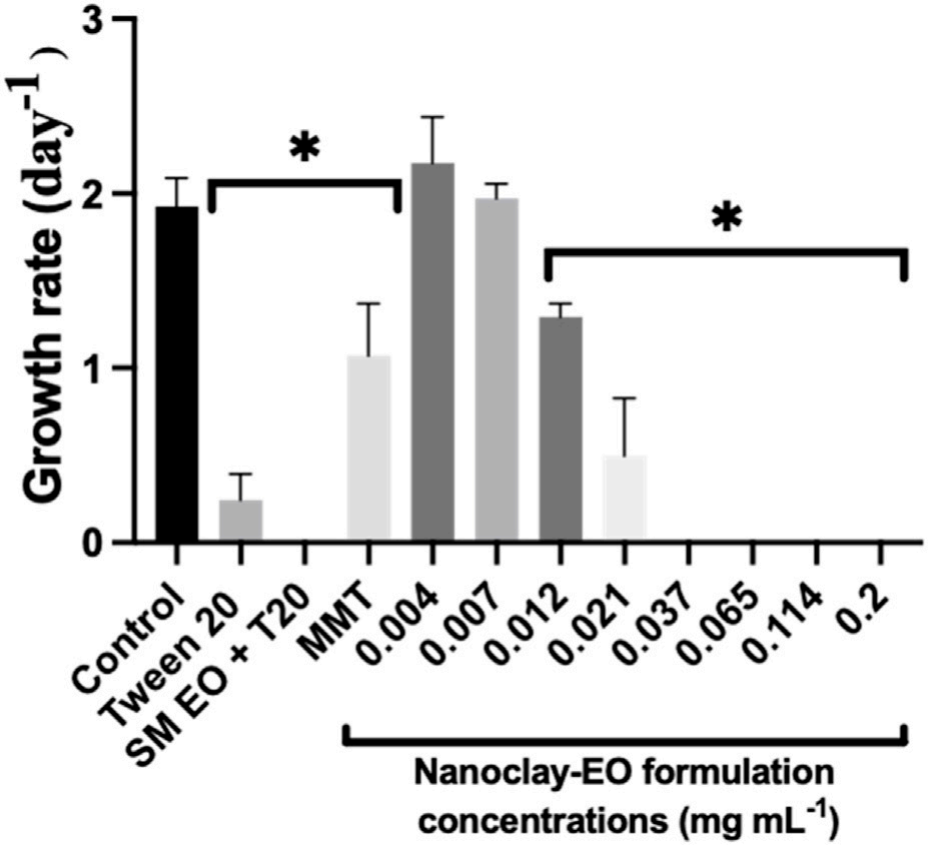
Average growth rate of *R. subcapitata* microalgae after 72 h of exposure to increasing concentrations of the nanoclay-EO formulation and to each one of its individual components highest concentration (SM EO (0.2 mg mL^-1^), Tween 20^®^ (0.2 mg mL^-1^) and MMT (5  mg mL^-1^)) for 72 h. SM EO + T20-*S. montana* EO + Tween 20^®^; MMT- Montmorillonite. Error bars represent the standard deviation. Asterisks denote significant differences from the control (p < 0.05).

In what regards, the microalgae inhibition test, analysing the results presented in Figure 2 it can be observed that all the components of the formulation were toxic to *R. subcapitata* with the *S. montana* EO + Tween 20^®^ presenting the highest toxicity, followed by Tween 20^®^ and MMT. These findings are in agreement with those reported by Wang et al. (2023), which also observed that the exposure of the macroalgae *Cladophora glomerata* to a mixture of EO extracted from different plants species (e.g., thyme, eucalyptus, perilla, clove and mint) at a maximum concentration of 0.3 mL mL^-1^ caused significant effects on cell morphology, photosynthetic system, oxidative stress and induced programmed cell death through increased caspase-like (3,8,9) activity (Wang et al., 2023). A study by Duringer et al. (2010), reported that EO from *Juniperus occidentalis* and *Chamaecyparis lawsoniana* had little to no risk to *R. subcapitata*, with NOEC values of 0.63 × 10^−3^ mg mL^-1^ for both oils, but the concentrations tested were lower than those tested in the present study.

Regarding the nanoclay-EO formulation, only the two lowest concentrations (0.004 and 0.007 mg mL^-1^) exhibited no toxicity toward the microalgae, with growth rates comparable to the control group. In contrast, the microalgae growth was totally inhibited for concentrations above 0.021 mg mL^-1^.

**FIGURE 3:**
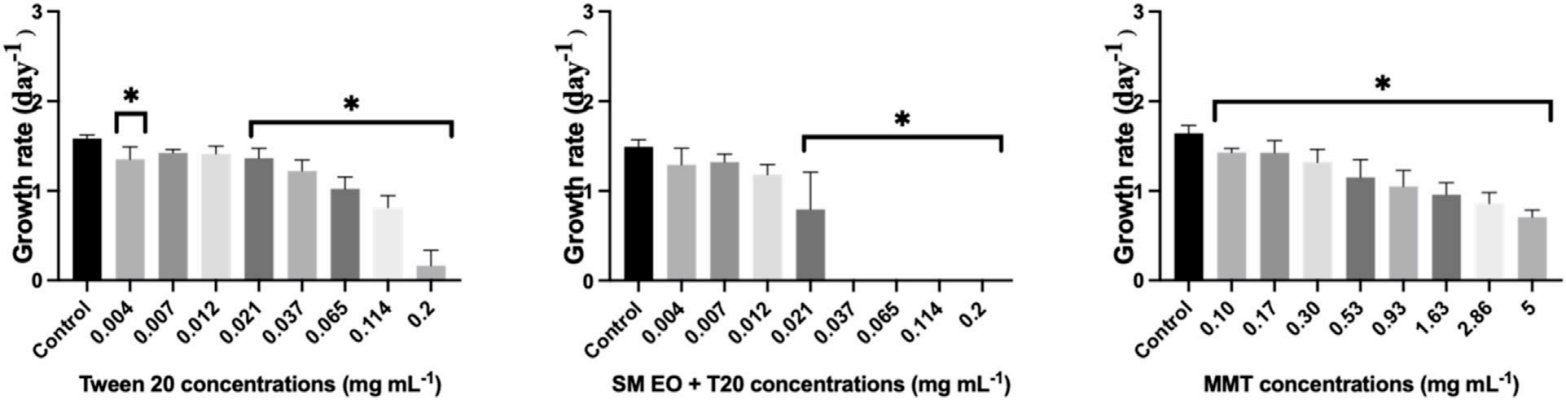
Average growth rate of the microalgae *R. subcapitata* after 72 h of exposure to increasing concentrations of the nanoclay-EO formulation individual components (EO (0.2 mg mL^-1^), Tween 20^®^ (0.2 mg mL^-1^), MMT (5 mg mL^-1^)). SM EO + T20-*S. montana* EO + Tween 20^®^; MMT-Montmorillonite. Error bars represent the standard deviation. Asterisks denote significant differences between the treatments and the control (level of significance p < 0.05).

To better assess the individual effect of Tween 20^*®*^, *S. montana* EO and MMT, on the inhibition *R. subcapitata* growth rate, the microalgae were exposed to concentrations of each nanoformulation component found at each concentration of the nanoclay-EO formulation tested (Figure 3). This approach followed in this study, enables a more accurate evaluation of the contribution of each component to the formulation´s overall toxicity. The results showed that the nano-clay formulation did not reduce the SM EO toxicity since the NOEC value of SM EO (0.012 mg mL^-1^) (dispersed in Tween 20^*®*^) was slightly higher than that of the nanoclay-EO formulation (0.007 mg mL^-1^). Also, if the observed effect at the lowest concentration of Tween 20^*®*^, was observed by chance, MMT was the most toxic component to the microalgae, likely contributing significantly to the nanoformulation´s toxicity. The MMT toxicity to the microalgae cells may be attributed to MMT’s properties that impact the microalgae’s capacity to absorb nutrients and to photosynthesize. These include its strong affinity for binding inorganic nutrients or for binding to cell walls surface, causing nutrients deficiency, or even by creating shading effects on microalgae cells (Tullio and Chalcraft, 2020). Nutrients such as nitrates and phosphates, vital for microalgae growth, can be adsorbed by the nanoclay, thereby reducing their availability (Fomina and Skorochod, 2020). Additionally, MMT can increase water turbidity, limiting light penetration necessary for photosynthesis (Tullio and Chalcraft, 2020). Moreover, according to Sultana et al. (2023), MMT may also act as potential flocculant making algae to flocculate and this process might have contributed to the algae’s poor growth, as it limits their accessibility to light (Sultana et al., 2023). A phenomenon that was also observed in our study. Collectively, these mechanisms may explain the poor growth of the microalgae.

**FIGURE 4:**
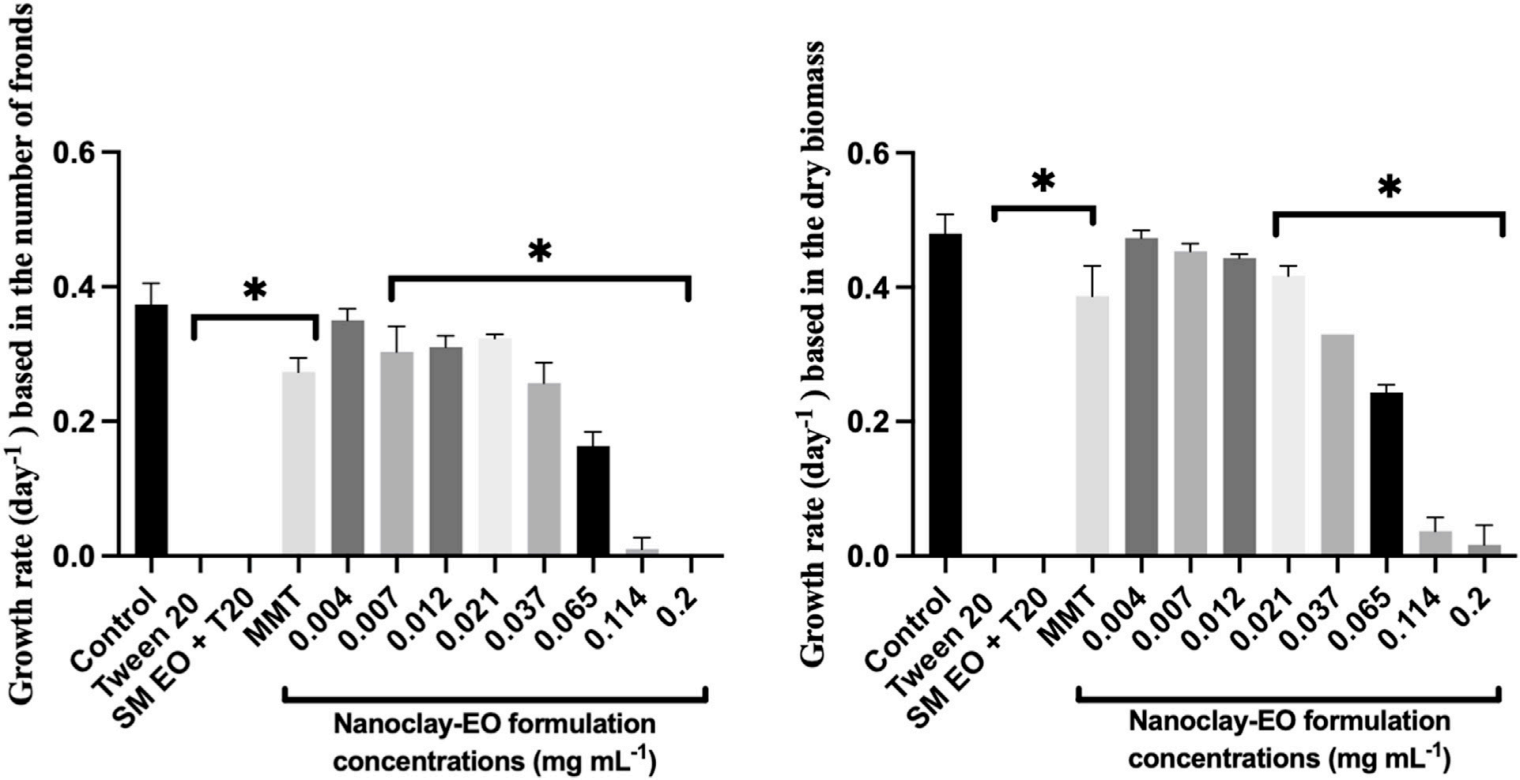
Average growth rate of *Lemna minor* based on the number of fronds (left) and dry biomass (right) after 7 days exposure to increasing concentrations of the nanoclay-EO formulation and the highest concentration of its individual components [(EO (0.2 mg mL^-1^), Tween 20^®^ (0.2 mg mL^-1^), MMT (5 mg mL^-1^)] for 7 days. SM EO + T20-*S. montana* EO + Tween 20^®^; MMT- Montmorillonite. Error bars represent the standard deviation. Asterisks denote significant differences between the treatments and the control (level of significance p < 0.05).

When the effect of the nanoclay-EO formulation was evaluated on *Lemna* minor, a severe toxic effect was observed for the three highest concentrations tested. The most sensitive parameter was the number of fronds, which may have resulted from physical interaction between the nanoclay and the plant’s fronds and subsequent effects on physiological processes. As explained above, nanoclay may interfere with light absorption and gas exchange by adhering to leaves and roots or by increasing water turbidity, which can impact both photosynthesis and plant growth (Gubbins et al., 2011; Tarrahi et al., 2018). Furthermore, both Tween 20^*®*^ alone and the EO dispersed on the surfactant, completely inhibited the growth of the macrophyte at the highest concentration (0.2 mg mL^-1^) tested, as assessed by the number of fronds and the dry biomass (Figure 4). The number of fronds was the most sensitive parameter to the nanoformulation.

**FIGURE 5:**
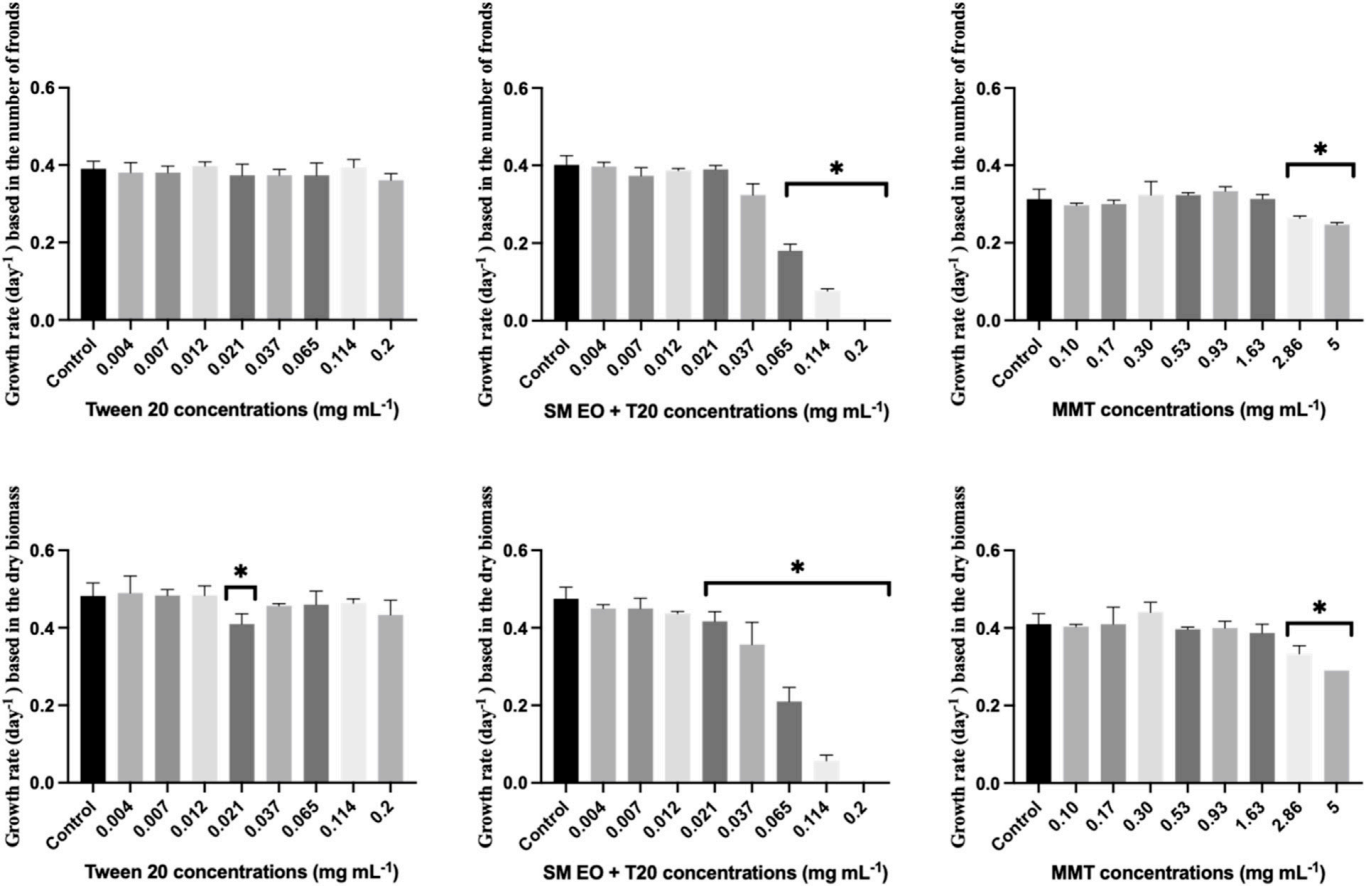
Average growth rate of *Lemna minor* based on the number of fronds (up) and dry biomass (down) after 7 days exposure to increasing concentrations of the individual components of the nanoclay-EO formulation (EO (0.2 mg mL^-1^), Tween 20^®^ (0.2 mg mL^-1^), MMT (5 mg mL^-1^)) for 7 days. SM EO + T20-*S. montana* EO + Tween 20^®^; MMT- Montmorillonite. Error bars represent the standard deviation. Asterisks represent significant differences between the treatments and the control (level of significance p < 0.05).

In addition, to evaluate the dose-dependent effect of the formulation´s individual components, *L*. *minor* was exposed to increasing concentration of the emulsifier (Tween 20^*®*^), emulsified SM EO and the nanoclay (MMT) (Figure 5).

As regards the effect of Tween20^®^ alone on *L. minor*, no toxicity was recorded for the concentrations tested. The significant reduction of plant growth rate registered at 0.021 mg mL^-1^ concentration of the surfactant; it is assumed to have occurred by chance as no toxic effect was observed for higher concentrations. In contrast, the *S. montana* EO emulsified with Tween 20^®^ showed a higher toxicity profile, with consistent and significant negative effects on growth rate for concentrations ≥0.021 mg mL^-1^, when growth rate was calculated based on dry biomass. According to the literature, *L. minor* has been used as test species in a study evaluating the toxicity of *Tanacetum macrophyllum* EO which showed toxicity at 10 mg mL^-1^, a concentration much higher than the ones tested in this study (Polatoğlu et al., 2015). Further, this EO is rich in sesquiterpenes, including copaborneol, γ-eudesmol, and (E)-sesquilavandulol, which are not present in the SM EO. In another study, using four different EO incorporated into chitosan nanoparticles, *S. montana* EO was among the most toxic, likely due to the presence of carvacrol and thymol in its chemical composition (authors unpublished data).

**FIGURE 6:**
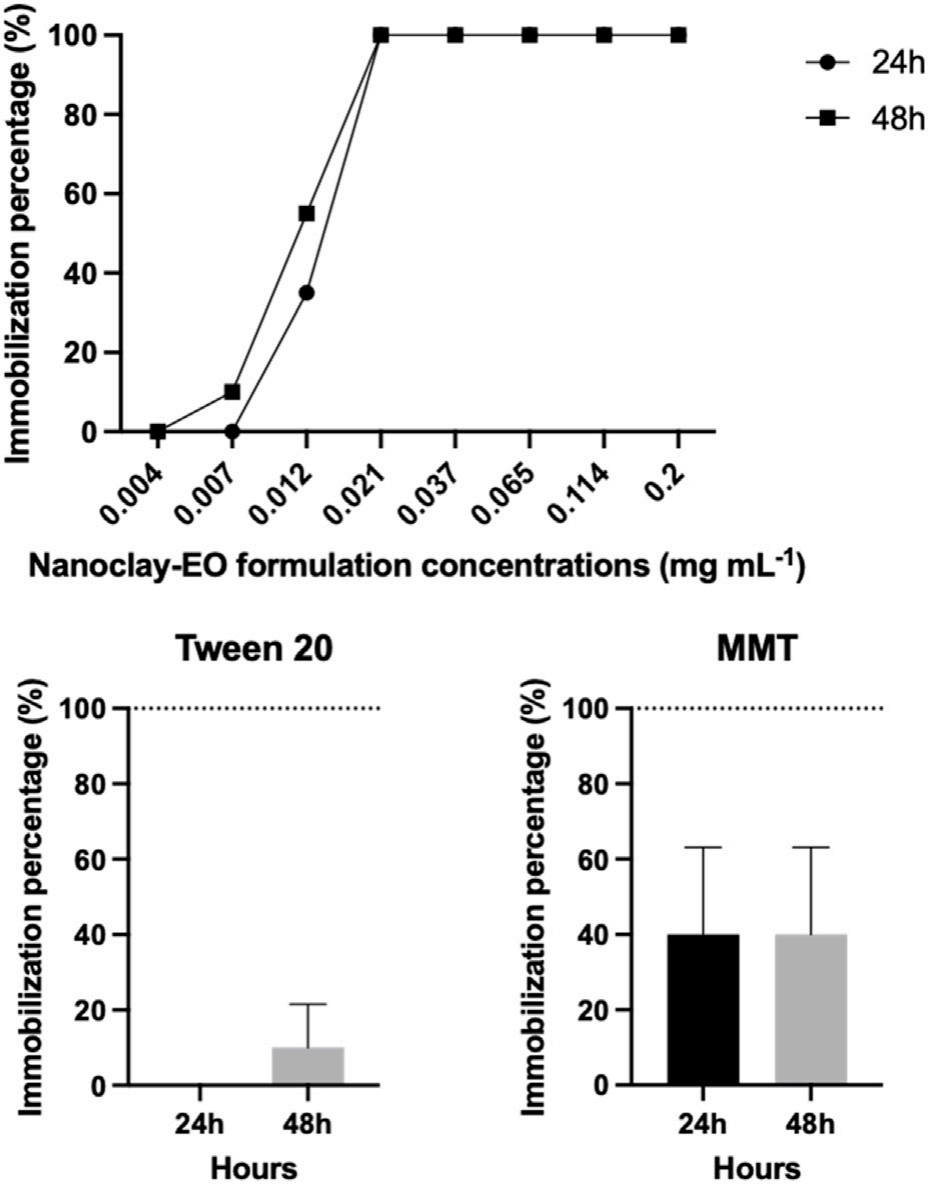
Average immobilization percentage of the microcrustacean *Daphnia magna* after 48 h of exposure to increasing concentrations of the nanoclay-EO formulation and to each one of its individual components (Tween 20^*®*^ (0.2 mg mL^-1^) and MMT (5 mg mL^-1^)) at the highest concentration present in nanoclay-EO formulation. Immobilization results for SM EO + Tween20^®^ are not shown, as 100% immobilization occurred in the first minutes. Error bars represent standard deviation.

The effect of the nanoclay-EO formulation on *D. magna* was also evaluated, through an immobilization test. There was an increase in *Daphnia magna* immobilization percentage with increasing concentrations of the nanoclay-EO formulation with 100% immobilization being observed for concentrations equal to or higher than the 0.021 mg mL^-1^ (Figure 6). A similar trend was observed for 24 and 48 h of exposure, with an EC_50_ = 0.011 mg mL^-1^ for 48 h of exposure. When the components were tested individually, there was a 10% immobilization on daphnids exposed to Tween 20^*®*^ (0.2 mg mL^-1^) and 40% immobilization when exposed to MMT (5  mg mL^-1^), after 48 h of exposure. Is noteworthy that all cladocerans exposed to SM EO + Tween 20^*®*^ became immobile within minutes (data not shown), identifying the EO as the primary source of toxicity. For this reason, the exposure to the range of concentrations of each one of the components was not performed.

These findings are in accordance with previous studies where a great toxicity to *D. magna* was observed for different EO. For example, Duringer et al. (2010) reported acute toxic effects of *C. lawsoniana* EO on this species with a EC_50_ = 1.9 × 10^−3^ mg mL^-1^ at the end of 48 h (Duringer et al., 2010). Zanfirescu et al. (2020) also observed an acute toxic effect of EO derived from the aerial portions of *Achillea millefolium* and of the female cones of *Taxodium distichum* with LC_50_ of 1.36 10^−3^ and of 1.09 × 10^−3^ mg mL^-1^, respectively, at the end of 48 h (Seo et al., 2012; Zanfirescu et al., 2020). In another study Seo et al. (2012) tested the effect of *Trachyspermum ammi* EO (chemically similar to SM EO, containing thymol, γ-terpinene, and p-cymene) on *D. magna* and reported a 48 h-EC_50_ value of 8.53 × 10^−3^ mg mL^-1^ (Seo et al., 2012). These concentrations are much lower than those used in the present study, reinforcing the high toxicity of EO extracted from different plant species to cladocerans. Additionally, MMT likely contributed to the toxicity of the nanoclay-EO formulation by clogging the gut tract, reducing food uptake and assimilation (Robinson et al., 2010).

As observed with the other two aquatic organisms, *R. subcapitata* and *L. minor*, the SM EO was the main contributor for the toxicity of the nanoformulation, alongside MMT.

Regarding the aquatic organisms tested, *R. subcapitata* and *D. magna* were the most sensitive. An interesting observation was made regarding *L. minor*: the nanoclay-EO formulation presented a NOEC of 0.012 mg mL^-1^ based on dry biomass, showing that MMT nanoclay did not reduce the toxicity of EO (NOEC = 0.012 mg mL^-1^) to the macrophyte, which was less sensitive than the microalgae, in what regards this parameter. However, when considering NOEC results based on the number of fronds, *L. minor* was more sensitive (NOEC = 0.004 mg mL^-1^) even than the microalgae (NOEC = 0.007 mg mL^-1^). This suggests that the loss of fronds may have been a strategy of the macrophyte to try to tolerate induced toxicity (Topp et al., 2011).

### 4.2 Ecotoxicological evaluation on the terrestrial organism

**FIGURE 7:**
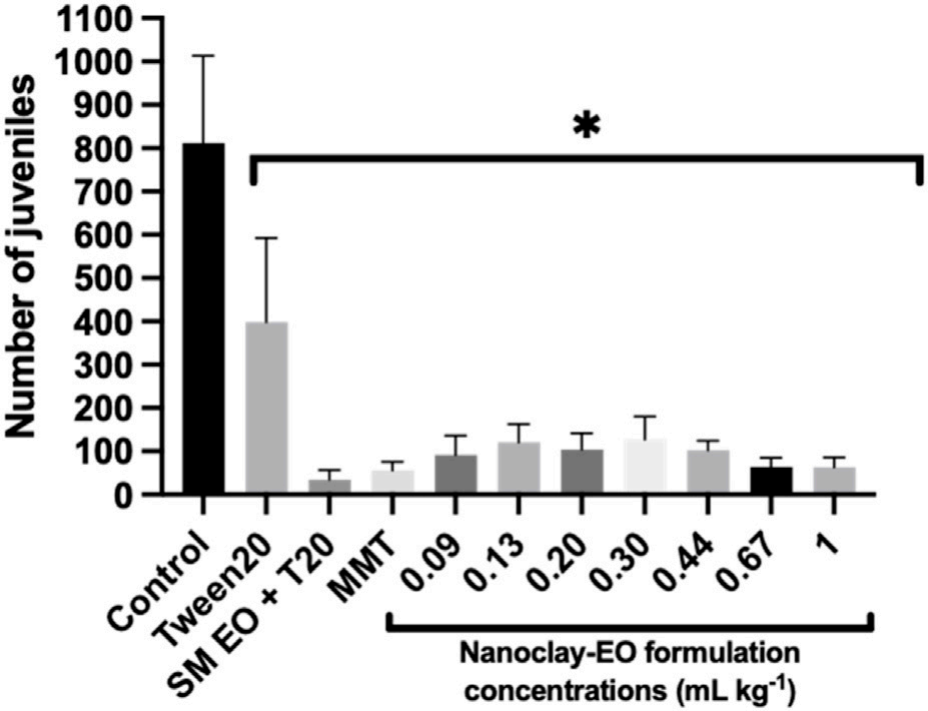
Average number of juveniles produced by *Folsomia candida* adults after 28 days of exposure to increasing concentrations of the nanoclay-EO formulation and to each one of its individual components ((EO (0.2 mg mL^-1^), Tween 20^®^ (0.2 mg mL^-1^), MMT (5 mg mL^-1^)). SM EO + T20- *S. montana* EO + Tween 20^®^; MMT- Montmorillonite. Error bars represent standard deviation. Asterisks represent significant differences between the treatments and the control (level of significance p < 0.05).

The reproduction of the collembolan *Folsomia candida* was significantly affected by the nanoclay-EO formulation, with a marked reduction in offspring observed for all the concentrations tested, when compared to control group (soil spiked only with water) (Figure 7).

**FIGURE 8:**
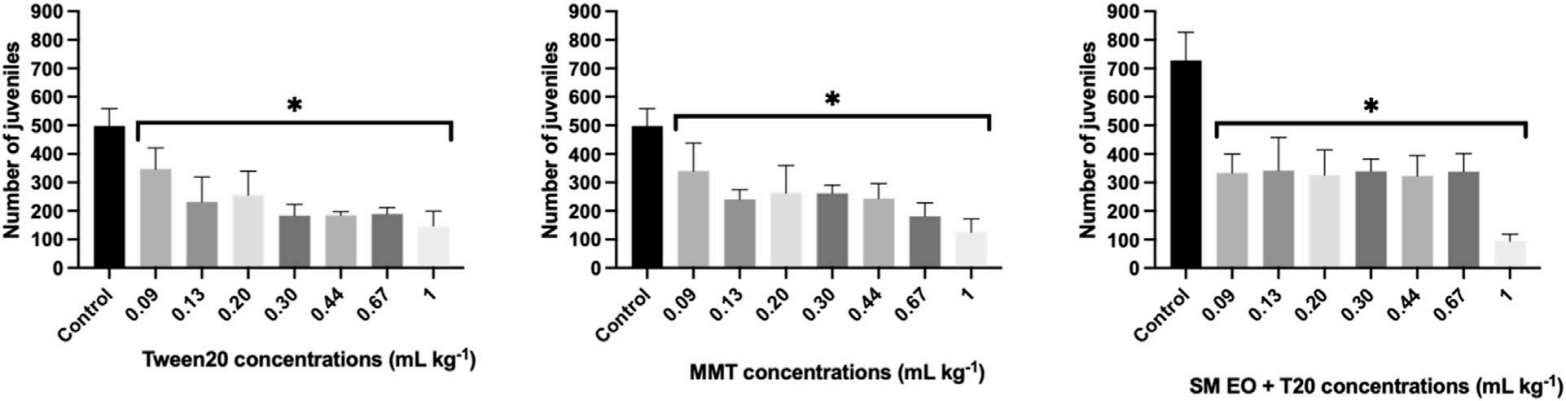
Average number of juveniles produced by *Folsomia candida* adults after 28 days exposure to increasing concentrations of the nanoclay-EO formulation individual components ((EO (0.2 mg mL^-1^), Tween 20^®^ (0.2 mg mL^-1^), MMT (5 mg mL^-1^)). SM EO + T20-*S. montana* EO + Tween 20^®^; MMT- Montmorillonite. Error bars represent the standard deviation. Asterisks represent significant differences between the treatments and the control (level of significance p < 0.05).

Springtails were also used for testing the dose effect of the components of the formulation on reproduction (Figure 8).

These results are consistent with previous findings in this study using aquatic organisms, demonstrating that all components of the nanoclay-EO formulation contributed, at least partially. To the best of our knowledge, the toxicity of MMT clay to collembola was never tested previously, as this mineralogical material is often considered a natural and inert material, with no expected toxic effects. Regarding the toxicity of EO to collembola, a study with *Eucaliptus globulus* EO revealed that it had both indirect impacts on the quality and availability of food for soil invertebrates, as well as direct harmful effects on soil fauna (EC_50_ of 35 mg kg^-1^ was reported to *F. candida*) (Martins et al., 2013). Additionally, when cinnamon EO was tested on springtails, Volpato et al. (2016) found that the concentration of 10 mg kg^-1^ significantly affected collembola reproduction compared to the control group (p < 0.05) (Volpato et al., 2016). In the present study, the *S. montana* EO caused toxic effects at much lower concentrations, likely due to its different chemical composition, suggesting the high toxicity of this EO to springtails. It cannot be ignored, as well, that the test was performed in the artificial OECD soil, a laboratorial prepared soil which has a poor microbial community. This may also have accounted for the highest toxicity of the EO and of the nanoclay EO formulation, despite the lower concentrations tested, as this soil has a lower capacity to mineralize organic toxic compounds. Overall, these findings align with the limited available literature, confirming that plant-derived EO can be toxic to soil invertebrates. This study provided valuable ecotoxicological data to assess the potential environmental risks of plant-based substances prior to their application as biopesticides. Furthermore, it was demonstrated that the nanoformulation does not reduce the toxicity of SM EO as a bioactive compound to non-target aquatic and terrestrial species. A summary of all ecotoxicological data obtained in this study is presented in Supplementary Table S3.

### 4.3 Soil microbial parameters

**FIGURE 9:**
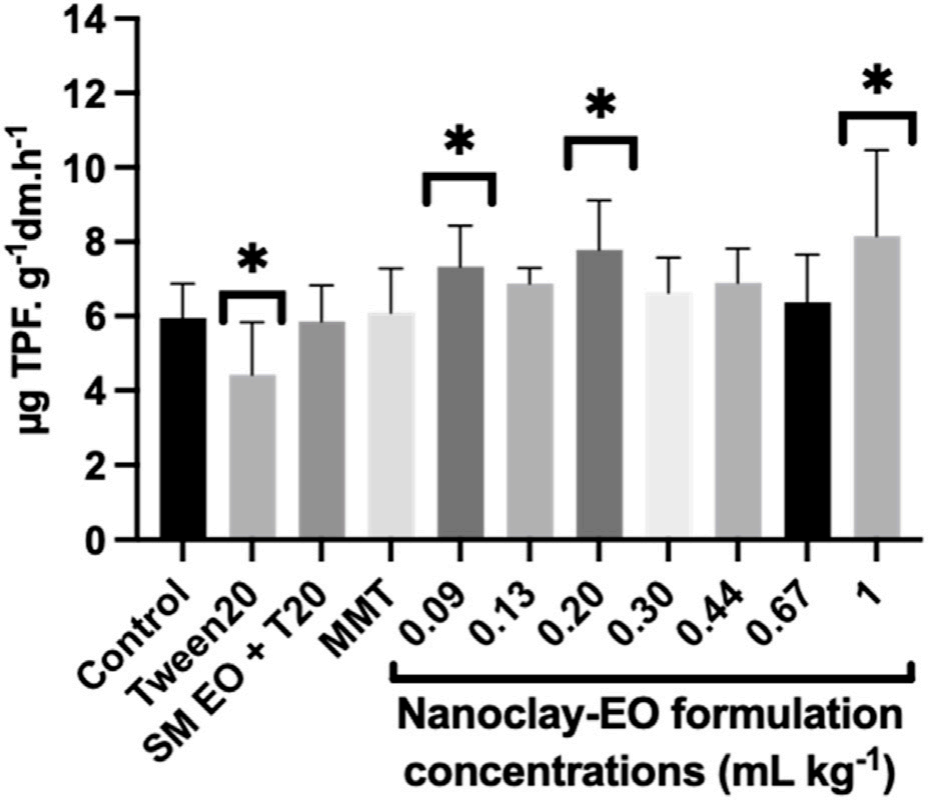
Average soil dehydrogenases activity after 30 days of exposure to a range of concentrations of the nanoclay-EO formulation and to each one of its individual components highest concentration ((SM EO (0.2 mg mL^-1^), Tween 20^®^ (0.2 mg mL^-1^), MMT (5 mg mL^-1^)). SM EO + T20- *S. montana* EO + Tween 20^®^; MMT- Montmorillonite. Error bars represent the standard deviation. Asterisks represent significant differences from the control (p < 0.05).

Soil dehydrogenases activity is an excellent indicator of overall metabolic activity of a vital soil microbial community as these enzymes are part of respiratory chain of microbial cells (Doi and Ranamukhaarachchi, 2009). The addition of organic sources, if not toxic, could enhance the amount of soil substrate that the microbial population can mineralize, as expressed by an increased activity of dehydrogenases (Adak et al., 2014). Therefore, this can explain the stimulatory effect observed, at least at some concentrations of the nanoclay-EO formulation tested, since the EO can act as a natural carbon source for the microbial community (Figure 9). Moreover, the presence of phenols in *S. montana* EO can also explain the stimulatory effect seen for this enzyme activity, which was previously noted as well by Zhou and Wu, (2012). When compared to the soil only moisturized with water (1.21 ± 0.04 µg TPF g^-1^ soil 24 h^-1^), the dehydrogenase activity in the same soil, containing a mixture of phenolic compounds, showed a significantly higher dehydrogenase activity (2.93 ± 0.22 µg TPF g^-1^ soil 24 h^-1^). Additionally, Chalkos et al. (2021) also found that the enrichment of soils with different textures, from various ecosystems (oak forest, mixed deciduous forest, riparian forest, phrygana, sandy shore and desert), with monoterpenes - the major constituents of SM EO - increased soil respiration and bacterial community density (Chalkos et al., 2021). Our results were also consistent with previous studies, which observed an increase in soil respiration under the exposure to EO with different chemical composition (Vokou and Liotiri, 1999; Vokou et al., 2002) and to different EO components (Vokou and Margaris, 1988). Vokou et al. (2002) also concluded that this enhancement was mainly a primary effect, resulting from the use of EO as a carbon and energy source by tolerant bacteria (e.g., *Pseudomonas*). The increase in soil respiration was concomitantly observed by these authors with an enhancement of soil bacterial colonies.

The surfactant Tween20^*®*^ was the only component showing an inhibitory effect on dehydrogenase activity. Due to its amphiphilic nature, Tween 20^*®*^ may disrupt microbial cell membranes, leading to cell lysis or damage. This disruption can reduce the number of viable microorganisms that contribute to dehydrogenase activity or impair the electron transport systems, where dehydrogenases play a key role (Marinari et al., 2000; Nannipieri et al., 2003). Non-ionic surfactants like Tween 20^*®*^ have been also reported as interferents with soil enzymatic hydrolysis processes by affecting enzyme-substrate interactions. For instance, they may reduce enzyme adsorption onto substrates, thereby diminishing enzyme efficiency and activity in soil environments (Wang et al., 2020).

Because phosphorous (P) is an essential nutrient that is usually present in the soil in less accessible forms, phosphatases are activated to increase P availability (Khan et al., 2023). Through the cleavage of organic phosphorus molecules, the extracellular acid phosphatase enzymes, produced by bacteria, fungi and plant roots are essential to produce soil phosphate ions (PO_4_^3-^) to be absorbed by plants and other photosynthetic microorganisms (Sun et al., 2020).

**FIGURE 10:**
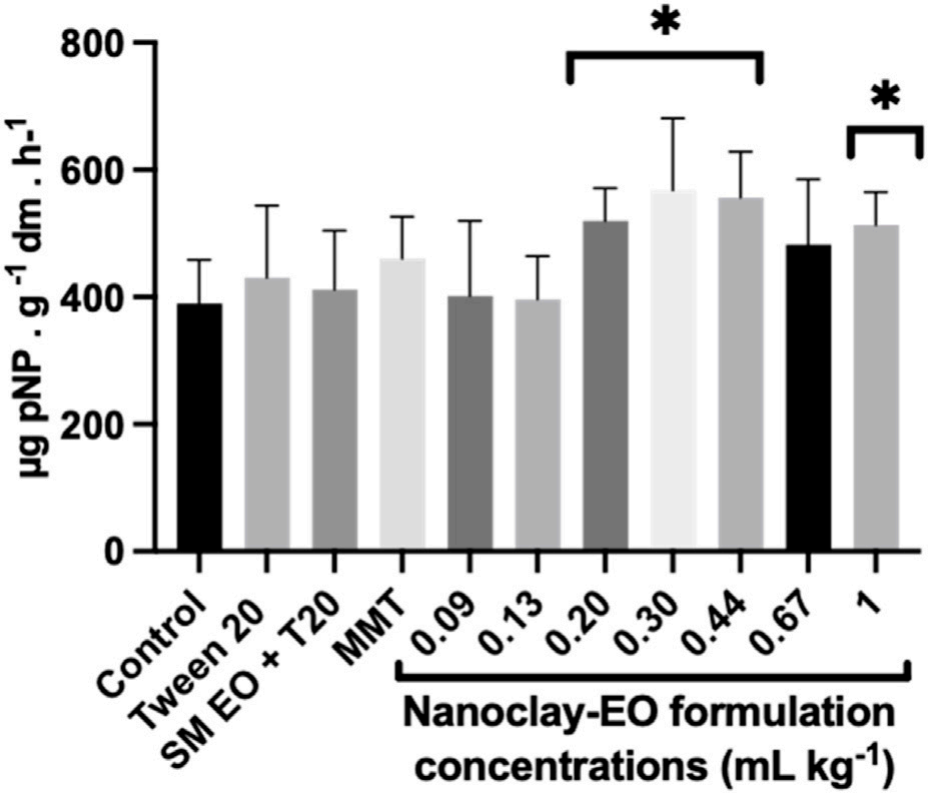
Acid phosphatase activity in soils exposed for 30 days to increasing concentrations of the nanoclay-EO formulation and to each one of its individual components highest concentration ((SM EO (0.2 mg mL^-1^), Tween 20^®^ (0.2 mg mL^-1^), MMT (5 mg mL^-1^)). SM EO + T20-*S. montana* EO + Tween 20^®^; MMT- Montmorillonite. Error bars represent the standard deviation. Asterisks represent significant differences between the treatments and the control (level of significance p < 0.05).

The nanoclay-EO formulation has stimulated the activity of these enzymes, with the highest concentrations showing the greatest stimulation effect (Figure 10). This increase may have been driven by the presence of the EO since it can act as a carbon source for soil microbes, which in turn release several enzymes to mine the nutrients needed for their development. In fact, this is the mainly supported hypothesis. The exposure to the individual components of the nanoclay did not cause any significant effect on the activity of acid phosphatases. And these results are not consistent with the results from previous studies that reported an inhibition of these enzymes under the exposure to EO (*E. citriodora*, *O. basilicum* and *M. arvensis*) (Khare et al., 2019) and to MMT (Shindo et al., 2002). The findings of this later study suggested that the adsorption of the enzyme to the MMT reduces its catalytic efficiency when compared to the free enzyme. Nevertheless, although these studies are relevant for understanding the mechanisms, the different experimental conditions limit the possibility of using it for interpreting our results.

The increased phosphatases activity observed may also have been a compensatory response to reduced P availability. Montmorillonite (MMT), like other clay minerals, has a known capacity to adsorb phosphorus, due to their lattice structure and high specific surface area immobilizing it, thereby decreasing its bioavailability (Uddin et al., 2017; Li et al., 2021; Chen and Arai, 2023). Therefore, the possible reduction in available P may have triggered microbial cells to enhance phosphatases production and release as a mechanism to mineralize organic P (García-Berumen et al., 2025). Nevertheless, and considering that the MMT alone did not cause such effect, this hypothesis is less supported, reinforcing the first proposed explanation based on the use of EO from the nanoformulation as an energy and carbon source, that increased the need of available phosphorus and other nutrients by the soil microbial community.

**FIGURE 11:**
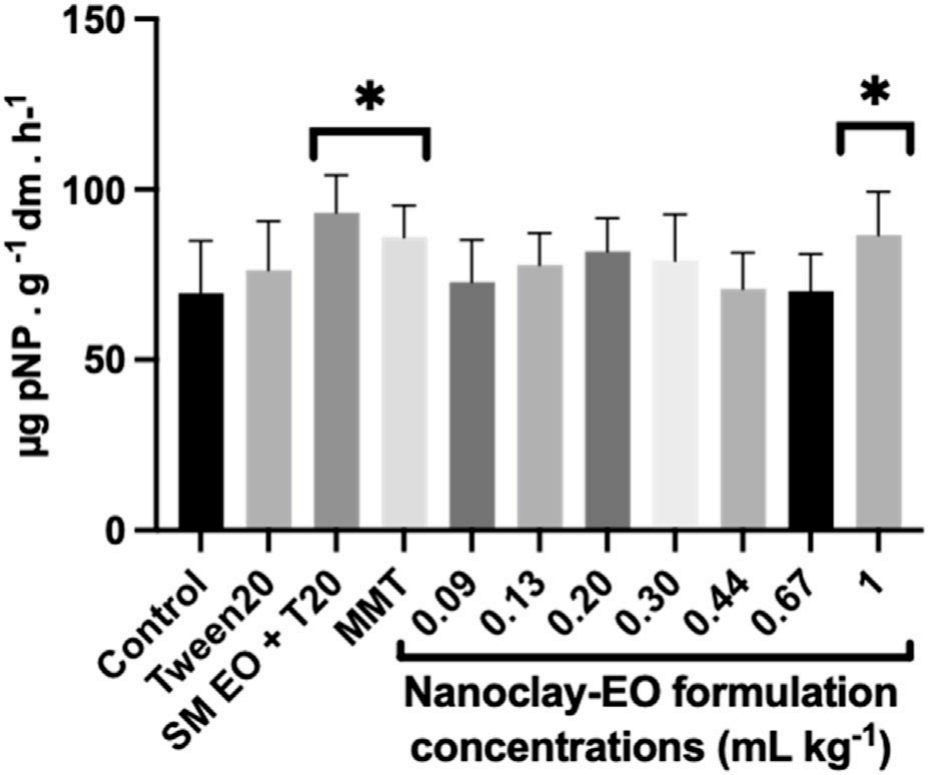
Arylsulfatase activity in soils exposed for 30 days to increasing concentrations of the nanoclay-EO formulation and to each one of its individual components highest concentration ((SM EO (0.2 mg mL^-1^), Tween 20^®^ (0.2 mg mL^-1^), MMT (5 mg mL^-1^)). SM EO + T20-*S. montana* EO + Tween 20^®^; MMT- Montmorillonite. Error bars represent standard deviation. Asterisks represent significant differences between the treatments and the control (level of significance p < 0.05).

The mineralization of sulphur-containing compounds in soils depends on sulfatases responsible for hydrolysing organic sulphates, which gives plants access to sulphur in an available form (SO_4_^2-^) (Robinson et al., 1997). By breaking the O-S-bond and catalysing the hydrolysis of arylsulfate anions, arylsulfatases regulate the mineralization of organic sulphur and, consequently, the sulphur cycle in the soil (Siwik-Ziomek et al., 2025). So, the availability and transformation of sulphur therefore depend on the activity of sulphur-mineralising microbial communities and their enzymatic output (Liu et al., 2025). Despite their ecological importance, arylsulfatases remain less studied in terms of their activity and regulation compared to other soil enzymes (Chen et al., 2019). In this study, the soils exposed to all concentrations of the nanoclay-EO formulation exhibited arylsulfatase activity levels comparable to the control, except for the highest concentration (Figure 11). Moreover, a significant stimulatory effect on this enzymatic activity was observed with SM EO and MMT exposure, at their highest concentration. As previously explained for other soil enzymes, the stimulatory effect was likely caused by the supplementary carbon source for soil microbes, offered by the EO, enhancing microbial metabolism and proliferation, which can lead to increased synthesis and activity of enzymes, including arylsulfatase (Chen et al., 2019; Vitanza et al., 2019). In parallel, montmorillonite, can adsorb and stabilize enzymes and organic compounds, protecting them from degradation and supporting microbial habitats (Chen and Arai, 2023). Although, this stabilization may affect the enzymatic activity, as previously explained, the synergistic interaction between the EO and MMT likely created a more favourable microenvironment for extracellular enzymes as arylsulfatases thereby enhancing enzyme activity in the soil. In fact, this stabilization effect may also have accounted for the stimulatory effects on other soil microbial parameters.

Microorganisms with different physiological traits are involved in the mineralization of organic nitrogen compounds. Organic molecules undergo ammonification, in which organic forms of nitrogen are reduced to ammonia (NH_4_^+^), and then oxidized to nitrate (NO_3_^−^) (Robinson et al., 1997).

**FIGURE 12:**
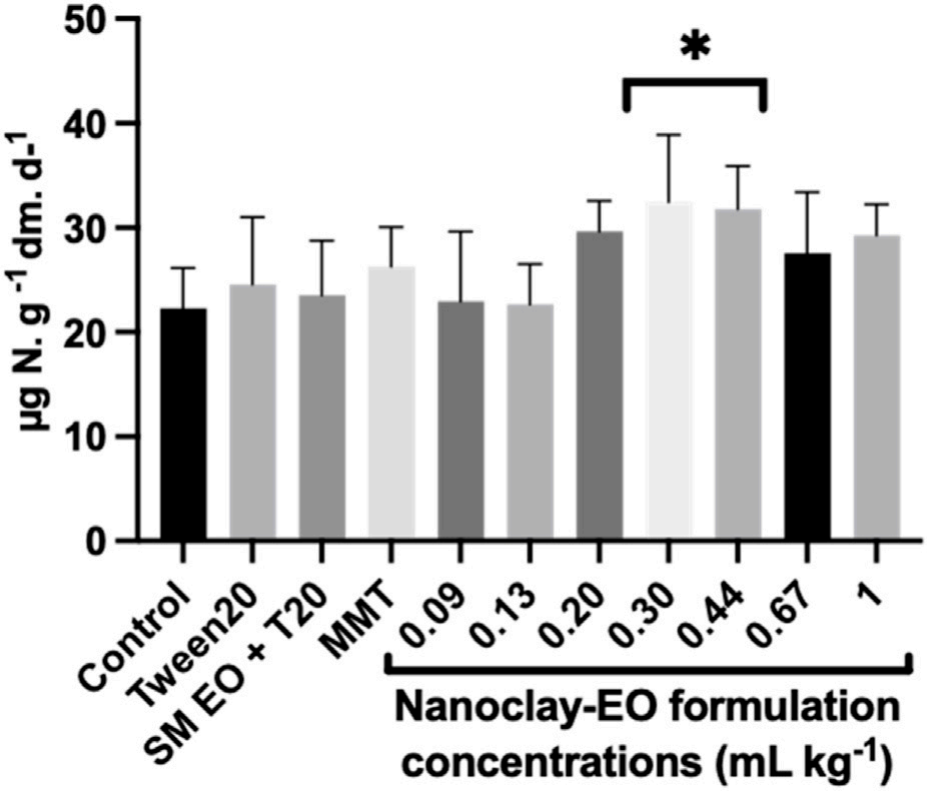
Nitrogen mineralization activity in soils exposed for 30 days to increasing concentrations of the nanoclay-EO formulation and to each one of its individual components highest concentration ((SM EO (0.2 mg mL^-1^), Tween 20^®^ (0.2 mg mL^-1^), MMT (5 mg mL^-1^)). SM EO + T20-*S. montana* EO + Tween 20^®^; MMT- Montmorillonite. Error bars represent standard deviation. Asterisks represent significant differences between the treatments and the control (level of significance p < 0.05).

As shown in Figure 12, the nanoclay-EO formulation did not significantly affect nitrogen mineralization at most tested concentrations. However, a significant stimulatory effect was observed at two of the highest concentrations tested, when compared to the control. This effect can be attributed to phenolic compounds present on SM EO, as phenolic acids have been shown to boost soil net N mineralization rates (Chen et al., 2020). Additionally, the stimulatory effect can also be due to the presence of MMT through the selective adsorption of low-molecular-weight and oxidized organic compounds onto the clay’s high-surface-area layers (Chotzen et al., 2016). By making these simpler substrates more bioavailable, montmorillonite enhances microbial access and enzymatic activity. Nevertheless, it is important to emphasize that the MMT alone did not influence this microbial parameter. Therefore, considering the increased dehydrogenase activity observed in this study—an indicator of enhanced soil microbial metabolic activity and organic matter degradation (Yang et al., 2023)—, these mineralization results suggest once again that heterotrophic soil bacteria were not negatively affected by the formulation, and possibly even benefit from it.

From a regulatory perspective, our results raise important concerns regarding the safe use of EO-based nanopesticides. The safe concentration for non-target species identified in this study (<0.007 mg mL^-1^) is markedly lower than the concentrations previously reported to be effective for pest control, such as ∼0.4 mg mL^-1^ against, for example, *Xanthomonas euvesicatoria* in tomato plants (Oliveira-Pinto et al., 2022b). This discrepancy indicates that the concentrations required to achieve efficacy in plant protection may exceed thresholds considered safe for aquatic and terrestrial non-target organisms. Such findings underscore the need for regulatory frameworks to carefully evaluate EO-based formulations, considering not only their biocidal potential but also their potential ecological risks, before authorization for widespread agricultural use.

## 5 Conclusion

The nanoclay-EO formulation was produced, nevertheless, it failed to reduce the toxicity of *S. montana* EO, as demonstrated by the ecotoxicological tests conducted with aquatic and terrestrial organisms. This could be explained by a possible less effective adsorption of the EO to the nanoclay. An aspect that must be analysed with more detail in a future study. The observed toxicity was caused mainly by the SM EO and the MMT. Among the tested organisms, the aquatic species *R. subcapitata* and *D*. *magna,* as well as the springtails *F. candida* showed to be highly sensitive to both the nanoformulation and its individual components, with significant negative impact observed at almost all tested concentrations. In a general way the soil microbial activity was less affected, as only stimulatory effects were registered for soil enzyme activities linked with the carbon, phosphorus and nitrogen cycles. Therefore, and despite EO have been suggested as potent anti-microbial agent, our results confirmed once again that the soil microbial community was able to deal with the EO-nanoclay formulation most likely by using it as a source of carbon and energy. Nevertheless, this does not mean that they did not caused changes in the overall composition of the soil microbial community, promoting the development of species tolerant to the EO. The results recorded for the soil enzymes activity leave room to hypothesize that ecotoxicological tests are probably overestimating the risks of EO, and EO based nanoformulations, considering that they are performed under axenic conditions or in a standard artificial soil, with a poor soil microbial community, that it is less able to mineralize organic compounds and to mitigate their toxicity. Despite that, results from previous studies also suggest that the ecotoxicity may also result from the SM EO, due to the presence of phenols (carvacrol and thymol) in its chemical composition, but also from MMT due to its mechanical but also adsorptive characteristics. Therefore, additional studies are required to better understand the ecotoxicological impacts of this EO-nanoclay formulation under real environmental scenarios and the broader ecological implications in soil ecosystems, resulting from the use of this formulation or other based-EO formulations as a nanopesticide. The impacts of cumulative applications also need to be assessed.

Despite these conclusions, this study generated data useful for the risk assessment of EO, following standard ecotoxicological tests and protocols, that are used for pesticides predictive risk assessment and registration. This study highlights that the current lack of knowledge about the risks associated with nanobiopesticides is particularly concerning, especially when remarkable negative effects are registered in species with relevant roles on terrestrial and freshwater trophic chains, despite the plant-based origin of some of its components. Based on current risk assessment approach of pesticides, that is mainly based on its active ingredient (Singh et al., 2024), a special attention should be given to the toxicity of EO, as they are being proposed as active ingredients of many nanobiopesticide formulations, and their combination with different types of nanomaterials do not seem to mitigate their toxicity, as some of them may also be toxic. Therefore, before testing nanobiopesticide formulations on target organisms to ascertain their efficacy as biopesticides, the safe dosages must be established to protect non-target species. Enhancing our understanding of nanobiopesticides ecotoxicology is essential to ensure their safe and sustainable application in agricultural practices.

## Data Availability

The authors selected the following statement: The original contributions presented in the study are included in the article/Supplementary Material, further inquiries can be directed to the corresponding author.
