# Supplementary material for: Montmorillonite-based essential oil carrier and its effects on non-target species: an environmental perspective on its risk assessment
Source: Front Toxicol. 2025 Oct 30;7:1696913. doi: 10.3389/ftox.2025.1696913 (PMC12611812; doi:10.3389/ftox.2025.1696913)
Supplement: Supplementary file 1 [file DataSheet1.docx]

**Supplementary Material**

**Table S1-** Conversion to W/W concentrations for the nanoclay-EO formulation and their individual components used in Folsomia candida reproduction test and the microbial parameters. The nanoclay-EO formulation concentrations are expressed based on the essential oil concentrations.

|  | **Concentrations (mL kg^-1^)** | **Concentrations (mg kg^-1^)** |
| --- | --- | --- |
| **Tween 20***^®^* | 1 | 0.40 |
| **MMT** | 1 | 10.00 |
| **SM EO + T20** | 1 | 0.40 |
| **Nanoclay-EO formulation** | 1 | 0.40 |
|  | 0.67 | 0.27 |
|  | 0.44 | 0.18 |
|  | 0.30 | 0.12 |
|  | 0.20 | 0.08 |
|  | 0.13 | 0.05 |
|  | 0.09 | 0.04 |

**Table S2-** Statistical results of the ecotoxicological tests performed to assess the effect of the nanoclay-EO formulation and its individual components.

| **Freshwater Microalgae Growth Inhibition Test** (p < 0.05) | |
| --- | --- |
| **Tween 20***^®^* | F (8.40) = 84.38 |
| **SM EO + T20** | Kruskal-Wallis = 44.78 |
| **MMT** | F (8.40) = 34.21 |
| **Nanoclay-EO formulation** | Kruskal-Wallis = 45.21 |
| **Lemna minor Growth Inhibition Test** (p < 0.05) | |
| **Tween 20***^®^* **(Fronds)** | Kruskal-Wallis = 9.83 |
| **Tween 20***^®^* **(Biomass)** | Kruskal-Wallis = 17.44 |
| **SM EO + T20 (Fronds)** | Kruskal-Wallis = 31.60 |
| **SM EO + T20 (Biomass)** | F (8.39) = 160.2 |
| **MMT (Fronds)** | Kruskal-Wallis = 22.38 |
| **MMT (Biomass)** | F (8.39) = 11.10 |
| **Nanoclay-EO formulation (Fronds)** | F (8.48) = 117.9 |
| **Nanoclay-EO formulation (Biomass)** | F (8.48) = 237.5 |
| ***Daphnia magna* Immobilization Test** | |
| **Nanoclay-EO formulation** | Chi-Square = 0.91 |
| ***F. candida* Reproduction Test** (p < 0.05) | |
| **Tween 20***^®^* | F (7.32) = 18.48 |
| **SM EO + T20** | F (7.32) = 15.68 |
| **MMT** | F (7.32) = 25.53 |
| **Nanoclay-EO formulation** | Kruskal-Wallis = 19.24 |
| **Soil Microbial Parameters** (p < 0.05) | |
| **Dehydrogenase** | |
| **Controls** | F (3.32) = 4.17 |
| **Nanoclay-EO formulation** | Kruskal-Wallis = 18.85 |
| **Acid phosphatase** | |
| **Controls** | F (3.32) = 1.05 |
| **Nanoclay-EO formulation** | Kruskal-Wallis = 34.40 |
| **Arylsulfatase** | |
| **Controls** | F (3.32) = 5.99 |
| **Nanoclay-EO formulation** | F (7.64) = 2.45 |
| **Nitrogen mineralization** | |
| **Controls** | F (3.32) = 1.05 |
| **Nanoclay-EO formulation** | Kruskal-Wallis = 34.40 |

**Table S3-** Summarized ecotoxicological information (NOEC or EC_x_) for the different test species exposed to the nanoclay-EO formulation and its individual components.

| **Microtox 81.9% Test** | | |
| --- | --- | --- |
|  | **EC_50_ (mg mL^-1^)** | **Highest Effect (%)** |
| **Tween 20***^®^* | - | 27 |
| **SM EO + T20** | <0.001 |  |
| **MMT** | - | 9.7 |
| **Nanoclay-EO formulation** | <0.001 |  |
| **Freshwater Microalgae Growth Inhibition Test** | | |
|  | **NOEC (mg mL^-1^)** | |
| **Tween 20***^®^* | 0.012 | |
| **SM EO + T20** | 0.012 | |
| **MMT** | <0.004 | |
| **Nanoclay-EO formulation** | 0.007 | |
| ***Lemna minor* Growth Inhibition Test** | | |
|  | **NOEC (mg mL^-1^)** | |
| **Tween 20***^®^* **(Fronds)** | >0.200 | |
| **Tween 20***^®^* **(Biomass)** | >0.200 | |
| **SM EO + T20 (Fronds)** | 0.037 | |
| **SM EO + T20 (Biomass)** | 0.012 | |
| **MMT (Fronds)** | 0.065 | |
| **MMT (Biomass)** | 0.065 | |
| **Nanoclay-EO formulation (Fronds)** | 0.004 | |
| **Nanoclay-EO formulation (Biomass)** | 0.012 | |
| ***Daphnia magna* Immobilization Test** | | |
|  | **EC_50_ (48h) (mg mL^-1^)** | |
| **Nanoclay-EO formulation** | 0.011 | |
| ***F. candida* Reproduction Test** | | |
|  | **NOEC (mL kg^-1^)** | |
| **Tween 20***^®^* | <0.090 | |
| **SM EO + T20** | <0.090 | |
| **MMT** | <0.090 | |
| **Nanoclay-EO formulation** | <0.090 | |
